# Melatonin Protects Against Mdivi-1-Induced Abnormal Spindle Assembly and Mitochondrial Superoxide Production During Porcine Oocyte Maturation

**DOI:** 10.3389/fcell.2021.693969

**Published:** 2021-07-08

**Authors:** Seul-Gi Yang, Seung-Yeon Joe, Jin-Wook Bae, Gyeong-Deok Heo, Hyo-Jin Park, Deog-Bon Koo

**Affiliations:** ^1^Department of Biotechnology, College of Engineering, Daegu University, Gyeongsan, South Korea; ^2^Institute of Infertility, Daegu University, Gyeongsan, South Korea

**Keywords:** melatonin, Mdivi-1, mitochondrial fission, spindle assembly, pig oocyte maturation

## Abstract

Mitochondrial division inhibitor 1 (Mdivi-1) reportedly provides a close connection between oocyte maturation and mitochondrial function in pigs. *N*-acetyl-5-methoxy-tryptamine (melatonin) is known to be a representative antioxidant with the ability to rehabilitate meiotic maturation of porcine oocytes. However, the ability of melatonin to recover Mdivi-1-mediated disruption of spindle formation during meiotic maturation of porcine oocytes during *in vitro* maturation (IVM) has not been studied. Here, we first investigated changes in mitochondrial length, such as fragmentation and elongation form, in mature porcine oocytes during IVM. Mature oocytes require appropriate mitochondrial fission for porcine oocyte maturation. We identified a dose-dependent reduction in meiotic maturation in porcine oocytes following Mdivi-1 treatment (50, 75, and 100 μM). We also confirmed changes in mitochondrial fission protein levels [dynamin-related protein 1 phosphorylation at serine 616 (pDRP1-Ser616) and dynamin-related protein 1 (DRP1)], mitochondrial membrane potential, and ATP production in 75 μM Mdivi-1-treated oocytes. As expected, Mdivi-1 significantly reduced mitochondrial function and DRP1 protein levels and increased spindle abnormalities in porcine oocytes. In addition, we confirmed that melatonin restores abnormal spindle assembly and reduces meiotic maturation rates by Mdivi-1 during porcine oocyte maturation. Interestingly, the expression levels of genes that reduce DNA damage and improve tubulin formation were enhanced during porcine meiotic maturation. Taken together, these results suggest that melatonin has direct beneficial effects on meiotic maturation through tubulin formation factors during porcine oocyte maturation.

## Introduction

In mammals, oocytes remain arrested at the first prophase of meiosis in meiotic maturation (Adhikari and Liu, 2014; Tukur et al., 2020). Abnormal spindle assembly connected to DNA damage likely occurs in oocytes during this extended period of arrest. In particular, spindle assembly plays an important role in exact chromosome congression and segregation during mammalian mitosis and meiosis (Yi et al., 2016). Spindle assembly occurs with chromosome separation in metaphase of the cell cycle (Bennabi et al., 2016). Correct chromosome separation is essential for mammalian oocyte maturation and subsequent fertilization (Hoshino, 2018). Damaged DNA is known to be associated with abnormal spindles during *in vitro* maturation (IVM) (Ganem and Pellman, 2012). *RAD51* and *γ-H2AX* are widely used as indicators of damaged DNA with DNA repair and damaged proteins, respectively (Mayer et al., 2016). Microtubules are known to be involved in various cellular events, including mitochondria distribution (López-Doménech et al., 2018), meiotic maturation (Lan et al., 2018), embryo development (Zenker et al., 2017), and spindle assembly (Romé and Ohkura, 2018). Spindle assembly relies on the expression of microtubule-related proteins (tubulin alpha 1a: *Tuba1a*, alpha-tubulin *N*-acetyltransferase: *Atat1*, and microtubule-associated protein 2: *Map2*), which are essential for microtubule stabilization in mammalian embryos (Namgoong and Kim, 2018; Sun et al., 2020). Microtubules are composed of α- and β-tubulin and undergo covalent modification, such as acetylation (Wloga et al., 2017). Furthermore, acetylated α-tubulin (AC-TUBULIN) as one of the tubulin formation factors plays a role in stabilizing microtubule structures for meiotic maturation and embryo development (Tang et al., 2018).

Mitochondria are the key regulators of metabolic homeostasis for cell survival, proliferation, differentiation, and division through adenosine triphosphate (ATP) production (Seo et al., 2018). A recent study showed that the mitochondrial dynamics response and morphological form changes are closely associated with mitochondrial function maintenance, regulation of reactive oxygen species (ROS) production, and cell death (Ježek et al., 2018). Mitochondrial dynamics is composed of fission and fusion, and maintaining its balance is associated with various centers of cellular and metabolic homeostases (Garone et al., 2018). Dynamin-related protein 1 (DRP1) as one of the mitochondrial fission factors is required for mitochondrial fission in mammals (Hu et al., 2017). Notably, DRP1 phosphorylation at serine 616 (pDRP1-ser616) directly induced mitochondrial fission (Park et al., 2019).

Mitochondrial division inhibitor 1 (Mdivi-1), a quinazolinone derivative, is a mitochondrial fission inhibitor (Otera et al., 2013) that influences mitochondrial morphology (Ruiz et al., 2018). Previous studies demonstrated that the maintenance of the homeostasis between mitochondrial fusion and fission is known to be important during oocyte maturation and early embryo development (Wakai et al., 2014; Yang et al., 2018). Disruption of this balance leads to mitochondrial dysfunction and apoptosis (Udagawa et al., 2014). Increasing mitochondrial fission by DRP1 generates new organelles and facilitates quality control (Youle and Van Der Bliek, 2012). However, mitochondrial fragmentation by excessive mitochondrial fission leads to *Cytochrome c* release and caspase activation (Bordt et al., 2017). DRP1 is an integral factor that is distributed in the cytoplasm and mitochondria for porcine oocyte maturation (Zhang et al., 2020). In addition, a previous study demonstrated that DRP1 overexpression causes oocyte maturation through abnormal spindle assembly in mouse oocytes (Wakai et al., 2014).

*N*-acetyl-5-methoxytryptamine (melatonin), an endogenous hormone produced by the pineal gland, is known to regulate circadian rhythms (Tan et al., 2016). Melatonin is well known to assist in improving the maturation capacity of oocytes and the development competence of preimplantation embryos through antioxidant effects in pigs (Yang et al., 2020). In addition, serotonin *N*-acetyltransferase (*Aanat*) is the first enzyme in the conversion of serotonin to melatonin (Schomerus and KORF, 2005). The mRNA expression of the *Aanat* gene is increased in oocytes during mouse oocyte maturation (He et al., 2016). Recently, it has been reported that mitochondria regulate melatonin synthesis and metabolism through *Aanat* expression in mouse oocytes (Han et al., 2017). However, no known studies have analyzed the *Aanat* gene expression pattern or its effects on mitochondrial fission in porcine oocytes. Moreover, melatonin is a well-known free radical scavenger in porcine embryos (Reiter et al., 2016) and protects against mitochondrial fission-mediated apoptosis (Ding et al., 2018).

Despite the well-known protective effects of melatonin on spindle assembly during meiotic maturation, the effect of melatonin on mitochondrial fission and fusion in porcine oocytes during IVM has not been investigated. Therefore, the objective of this study was to determine whether melatonin protects against abnormal spindle assembly in Mdivi-1-exposed porcine oocytes by blocking mitochondrial fission. We also found that melatonin restored spindle assembly damage induced by Mdivi-1, enhanced melatonin synthase capacity, enhanced the expression of factors related to tubulin formation, and subsequently improved meiotic maturation and oocyte quality in pigs. These findings provide the first evidence regarding the protective mechanisms on abnormal spindle assembly by Mdivi-1 connecting melatonin positive effects in porcine oocyte during IVM.

## Materials and Methods

### Chemicals

Unless otherwise noted, all chemicals in present study were purchased from Sigma Chemical Co. (St. Louis, MO, United States).

### *In vitro* Maturation

Porcine ovaries were acquired from non-pregnant sows in local abattoirs. They were conveyed to the laboratory in 0.9% saline with 75 μg/ml penicillin G at 38.5°C. Immature cumulus–oocyte complexes (COCs) were aspirated from 3- to 6-mm follicles using a 10-ml disposable syringe with an 18-gauge needle. Fifty immature COCs were matured in 500 μl of IVM medium at 38.5°C under 5% CO_2_. NCSU 23 medium with 10% follicular fluid, 0.57 mM cysteine, 10 ng/ml β-mercaptoethanol, 10 ng/ml epidermal growth factor (EGF), 10 IU/ml pregnant mare serum gonadotropin (PMSG), and 10 IU/ml human chorionic gonadotropin (hCG) were used for oocyte maturation. After culturing for 22 h, COCs were washed three times and then further cultured in maturation medium without PMSG and hCG for 22 h. Mdivi-1 (50, 75, and 100 μM) was added to the maturation medium during the IVM periods. For assessment of melatonin treatment, cells were pretreated with Mdivi-1 75 μM for 22 h, and then melatonin 100 nM was added into the medium for another 22 h.

### MitoTracker and Immunofluorescence Staining

Denuded oocytes were fixed in 3.7% formaldehyde overnight at 4°C. Fixed oocytes were incubated in IVM medium with 4 μM MitoTracker Green (Cell Signaling Technology, MA, United States) or 4 μM MitoTracker Orange (Invitrogen, CA, United States) for 30 min at 38.5°C. The intensity of MitoTracker images was acquired using LSM 800 confocal microscope (Zeiss, Jena, Germany) and iRiSTM Digital Cell Image System (Logos Biosystems, Gyeonggi-do, South Korea). Denuded oocytes were fixed in 3.7% formaldehyde in phosphate buffered saline (PBS) overnight at 4°C. Fixed oocytes were permeabilized in 0.5% Triton X-100 for 30 min at room temperature (RT). The oocytes were blocked with 1% bovine serum albumin (BSA) in polyvinyl alcohol (PVA) in PBS for 1 h at RT. Blocked oocytes were incubated with primary antibodies such as mouse monoclonal fluorescein isothiocyanate (FITC)-conjugated anti-α-tubulin antibody (1:100), anti-RAD51 (1:200 dilution; Santa Cruz Biotechnology, CA, United States), and γ-H2AX (1:200 dilution, Abcam, Cambridge, England) overnight at 4°C, and incubated oocytes were washed three times with 0.1% PVA in PBS. After incubating with primary antibodies, the oocytes were incubated with secondary antibodies, Alexa Fluor 488 goat anti mouse IgG (1:1,000 dilution) and Alexa Fluor 555 goat anti-rabbit IgG (1:1,000 dilution) (Thermo Scientific, MA, United States). Stained oocytes were mounted with 4′,6-diamidino-2-phenylindole (DAPI) solution on a glass slide. The intensity of α-tubulin and DAPI images was acquired using LSM 800 confocal microscope (Zeiss, Jena, Germany). We referred to the mitochondrial fluorescence image analysis method as described by Park et al. (2013). Mitochondria were divided into two different categories based on length ranging from less than 1 μm (fragmented form) and greater than 3 μm (elongation form). Mitochondrial aggregation in oocyte using MitoTracker green was analyzed as described by Soares et al. (2020). The mitochondrial aggregation pattern was measured by green fluorescence quantification using the ImageJ 1.46r software (NIH, United States) by the region of the oocyte cytoplasm. All images for analysis were taken using the same intensity and exposure time.

### Western Blot Analysis

Fifty denuded oocyte lysates in PRO-PREP protein lysis buffer (iNtRON, Daejeon, South Korea) were prepared. Oocyte lysates were separated by 10% sodium dodecyl sulfate polyacrylamide gel electrophoresis (SDS-PAGE) and transferred to pure nitrocellulose membranes (Pall Life Sciences, NY, United States) after electrophoresis. The membranes were incubated with primary antibodies such as anti-phospho-DRP1-Ser616 (Cell Signaling), DRP1 (Santa Cruz Biotechnology), AC-TUBULIN (Cell Signaling), and β-actin (Santa Cruz Biotechnology). The membranes were incubated with a secondary horseradish peroxidase (HRP)-conjugated anti-rabbit/mouse IgG (Thermo Scientific, MA, United States). Binding antibodies were detected using the enhanced chemiluminescence (ECL) kit (Bio-Rad, CA, United States). The bands were visualized using Fusion Solo software (Vilber Lourmat, Collégien, France).

### Immunohistochemistry

Porcine ovaries were fixed with 10% formalin solution. Fixed ovaries sliced into 3–5 μm sections were embedded in paraffin. The sections were deparaffinized and briefly heated before being treated with a protein lock solution (Dako, Carpinteria, CA, United States). And then, the sections were incubated with the anti-DRP1 (Santa Cruz Biotechnology). After being washed with 0.1 mol/l TBS containing 0.01% Tween-20 solution, the sections were incubated with anti-rabbit polymer (Dako). Peroxidases bound to the antibody complex were visualized by treatment with a 3,3′-diaminobenzidine (DAB) chromogen substrate solution (Dako). The DAB reaction was examined under a microscope to determine the optimal incubation time and was halted by washing several times with 0.1 mol/l TBS. The immunohistochemically labeled sections were dehydrated in a graded ethanol series, defatted in xylene, and mounted. The sections were observed using a microscope (Leica, Solms, Germany).

### Analysis of Meiotic Maturation, Intracellular Reactive Oxygen Species and Mitochondrial Superoxide

Denuded oocytes were fixed using acetic acid:ethanol (1:3) solution overnight at RT and then were stained with 2% orcein solution for 10 min. The meiotic maturation stage of each oocyte was confirmed under a microscope (Leica).

Denuded oocytes were incubated in IVM medium with 5 μM dichlorodihydrofluorescein diacetate (DCF-DA; Invitrogen) or 1 μM Mito-SOX red (Invitrogen) for 30 min at 38.5°C. The fluorescence images of DCF-DA and Mito-SOX were acquired using an LSM 800 confocal microscope (Zeiss).

### JC-1 Staining and ATP Determination

Denuded oocytes were incubated in IVM medium with JC-1 (100:1) (Cayman Chemical, MI, United States) for 30 min at 38.5°C. The stained oocytes were placed into 0.1% PVA in PBS drop of confocal dish under paraffin oil. The intensity of JC-1 images was acquired using iRiSTM Digital Cell Image System (Logos Biosystems). The concentration of ATP in denuded oocytes was measured using an ATP determination kit (Invitrogen). We made a standard reaction buffer according to the instructions of the manufacturer. Here, 50–60 oocytes were homogenized using 10 μl radioimmunoprecipitation assay (RIPA) buffer, and then 90 μl standard reaction buffer was added. The total mixed buffer was transferred 100 μl per well into a white 96-well plate. Luminescence intensity was measured using a luminometer (InfiniteM200pro, Tecan, Männedorf, Switzerland).

### Quantitative Real-Time PCR

RNeasy mini kit (Qiagen, Hilden, Germany) was used to extract total RNA from denuded oocytes. Quantitative polymerase chain reaction (qPCR) was performed with the TOPreal^TM^ qPCR 2X Premix (SYBR Green with low ROX) containing specific primers (Table 1) in a LightCycler^®^ 96 real-time fluorescent quantitative PCR machine (Roche, Basel, Switzerland). The PCR conditions were set as follows: 10 min at 95°C, followed by 45 cycles of 95°C for 10 s, 57–59°C for 10 s, and 72°C for 10 s. The expressions of genes were calculated using the 2^–ΔΔ*CT*^ method.

**TABLE 1:** Primer sequences and real-time PCR conditions in mature oocytes.

Genes	Description	Primer sequences	TM °C	Accession numbers	Base pairs
*Rad51*	Rad51 recombinase	For (5′–3′): TGGATGGAGCAGCCATGTTT Rev (5′–3′): TCCATCCGCATTGATGGCAA	59	NM_001123181.1	182 bp
*γ-H2ax*	H2a.x variant histone	For (5′–3′): TTAAATCTGGCGCGCTTCAC Rev (5′–3′): TTTAATACCCGCCTCCGGTT	58	XM_003129950.5	188 bp
*Aanat*	Aralkylamine *N*-Acetyltransferase	For (5′–3′): AGAAGCCTTCATCCCTGTCT Rev (5′–3′): TCAGCGACTCCTGAGTGATT	57	XM_005656910.3	170 bp
*Tuba1a*	Tubulin alpha 1a	For (5′–3′): AAATACATGGCCTGCTGCCT Rev (5′–3′): ATGCCAACCTTGAAGCCAGT	59	XM_005655549.2	134 bp
*Atat1*	Alpha tubulin acetyltransferase 1	For (5′–3′): TGTTGCAGAAGGAGCGAGTT Rev (5′–3′): TTGTTCACCTGTGGGACTGT	58	NM_001123123.1	118 bp
*Map2*	Microtubule associated protein 2	For (5′–3′): TGAAGCAAAGGCACCTCACT Rev (5′–3′): ATCCATTGGCGCTTCTGACA	58	XM_021075002.1	122 bp
*Gapdh*	Glyceraldehyde-3-phosphate dehydrogenase	For (5′–3′): GGAGAACGGGAAGCTTGTCA Rev (5′–3′): TTCACGACCATGGAGAAGGC	59	NM_001206359.1	138 bp

### Statistical Analysis

All the experiments in triplicate were presented as the obtained mean ± standard deviation (SD) or standard error of the mean (SEM). All results were analyzed using *t*-tests or one-way ANOVA followed Tukey multiple comparison test. All data were measured using the GraphPad Prism 5.0 software (San Diego, CA, United States). Histogram values of densitometry were evaluated by ImageJ 1.46r software (NIH). Differences were considered significant at ^∗^*p* < 0.05, ^∗∗^*p* < 0.01, ^∗∗∗^*p* < 0.001.

## Results

### Mitochondrial Fission Increases During Porcine Oocyte Maturation

**FIGURE 1:**
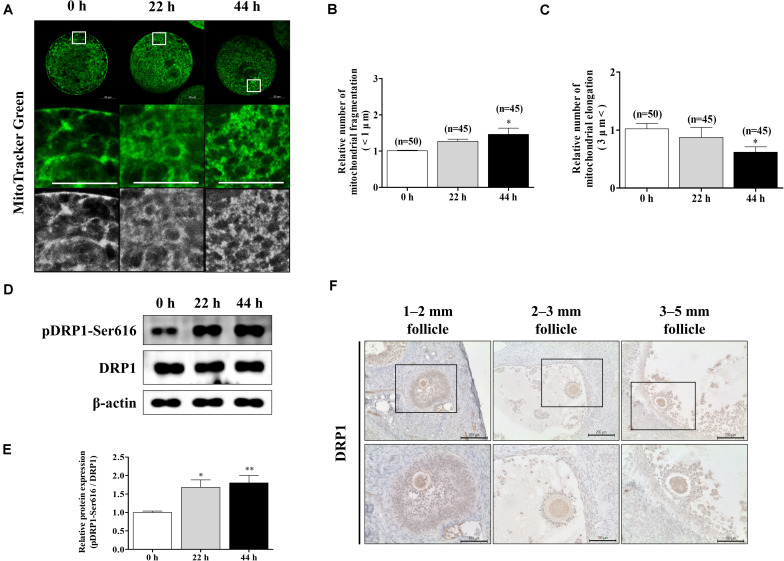
Changes of mitochondrial dynamics during meiotic maturation in porcine oocytes. **(A–C)** Porcine oocytes during meiotic maturation (0, 22, and 44 h) stained for mitochondrial fragmentation and elongation using MitoTracker green staining. Scale bar: 50 μm. **(D,E)** Representative Western blot and quantitative analyses of the expression of mitochondrial fission proteins [dynamin-related protein 1 phosphorylation at serine 616 (pDRP1-Ser616) and dynamin-related protein 1 (DRP1)] in immature and mature oocytes (0, 22, and 44 h). **(F)** Immunohistochemistry of DRP1 in various porcine ovarian follicles (1–2, 2–3, and 3–5 mm). Scale bar: 100 and 200 μm. Data in the bar graph represent the mean ± SD/SEM of three independent experiments (^∗^*p* < 0.05; ^∗∗^*p* < 0.01).

To confirm the changes in mitochondrial morphology during porcine oocyte maturation, we stained oocytes at 0, 22, and 44 h using MitoTracker Green staining (Figure 1A). Following oocyte maturation, the mitochondrial fragmentation (<1 μm) of oocytes significantly increased (Figure 1B). An opposite pattern of mitochondrial elongation (>3 μm) was found when oocytes were cultured at 44 h of IVM (Figure 1C). We investigated the expression patterns of mitochondrial fission-related proteins (pDRP1-Ser616 and DRP1) by Western blot analysis in porcine oocytes (Figure 1D). The protein level of pDRP1-Ser616 was significantly increased in mature oocytes (Figure 1E). Furthermore, we confirmed DRP1 protein levels using immunohistochemical staining in the ovary (follicle sizes 1–2, 2–3, and 3–5 mm; Figure 1F). DRP1 protein levels were detected in only oocyte cytoplasm from the ovary. Based on these results, we confirmed that mitochondrial fission continuously increased through a shortened mitochondria length and elevated pDRP1-Ser616 protein levels during porcine oocyte maturation.

### Mdivi-1 Interrupts Meiotic Maturation of Porcine Oocytes

**FIGURE 2:**
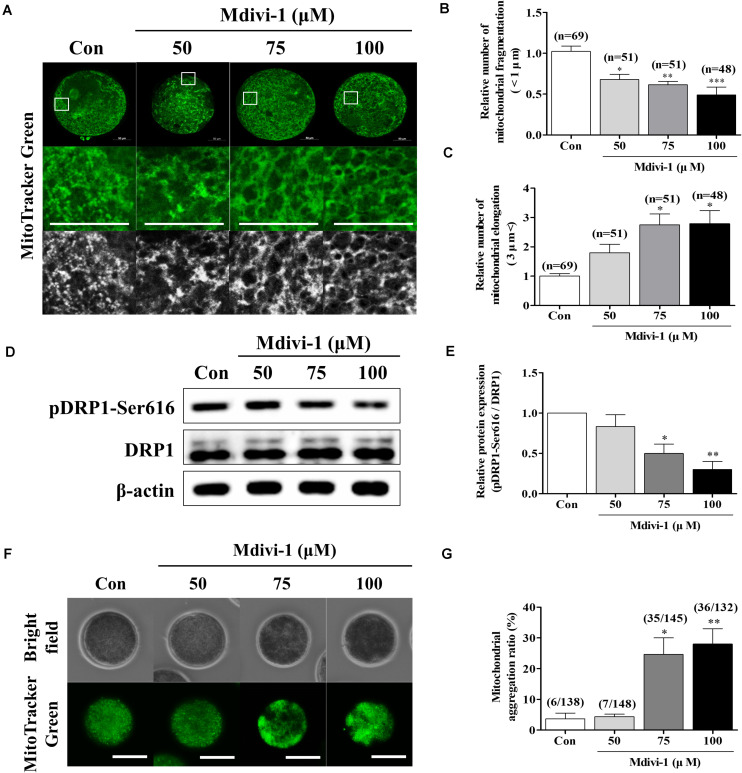
Effects of mitochondrial division inhibitor 1 (Mdivi-1) exposure on mitochondrial dynamics and distribution during meiotic maturation in porcine oocytes. **(A–C)** Representative images of mitochondrial length (fragmentation and elongation) in 0, 50, 75, or 100 μM Mdivi-1-treated oocytes. Scale bar: 50 μm. **(D,E)** Representative Western blot of the expression of mitochondrial fission proteins [dynamin-related protein 1 phosphorylation at serine 616 (pDRP1-Ser616) and dynamin-related protein 1 (DRP1)] in control and Mdivi-1-treated oocytes. Here, β-actin was used as a loading control. The ratios of phosphorylated/total proteins were calculated and plotted in all experimental groups. **(F,G)** Ratios of mitochondria aggregation were detected using MitoTracker green. Scale bar: 200 μm. Data represent the mean ± SD/SEM of three biological replicates (^∗^*p* < 0.05; ^∗∗^*p* < 0.01; ^∗∗∗^*p* < 0.001).

Increasing concentrations (50, 75, and 100 μM) of Mdivi-1, a mitochondrial fission inhibitor, were added to IVM medium for an *in vitro* oocyte culture to test whether Mdivi-1 treatment could interrupt meiotic maturation (Table 2). As a result, meiotic maturation significantly decreased in a dose-dependent manner (control: 77.9% ± 3.6% vs. 50 μM Mdivi-1: 59.5% ± 10.1%, 75 μM Mdivi-1: 54.1% ± 5.4%, and 100 μM Mdivi-1: 1.3% ± 2.3%). The number of fragmented mitochondria of porcine oocytes (<1 μm) continued to decline in a dose-dependent manner 44 h after IVM (Figures 2A,B). Mitochondrial elongation in the 75 and 100 μM Mdivi-1-treated group was significantly higher than that in the other groups (Figure 2C). As expected, pDRP1-Ser616 in Mdivi-1-treated oocytes significantly decreased in a dose-dependent manner after 44 h of IVM (Figures 2D,E). Mdivi-1 treatment in porcine oocytes did not significantly affect the total DRP1 protein levels. To observe the mitochondrial aggregation ratio in Mdivi-1-stimulated oocytes, we measured the percentage of oocytes with mitochondrial aggregation in oocytes from Mdivi-1-exposed groups. As a result, mitochondrial aggregation in oocytes by MitoTracker Green staining was increased in the Mdivi-1 treatment group at 44 h after IVM (Figures 2F,G). These results showed that Mdivi-1 disturbs porcine meiotic maturation by blocking mitochondrial fission during IVM.

**TABLE 2:** Meiotic maturation rate according to Mdivi-1 concentrations.

		% of oocytes (n)			
		
Mdivi-1 (μM)	No. of oocytes examined	GV	GVBD	M I	M II
Con	183	7.2 ± 3.6 (13)	2.4 ± 3.4 (4)	13.1 ± 2.6 (24)	77.9 ± 3.1 (142)^a^
50	189	6.1 ± 2.3 (11)	5.0 ± 2.0 (9)	30.0 ± 9.8 (54)	59.0 ± 8.3 (115)^b^
75	182	13.0 ± 10.5 (24)	8.1 ± 4.0 (15)	29.4 ± 4.9 (53)	49.6 ± 11.1 (90)^c^
100	158	44.8 ± 17.9 (75)	18.9 ± 14.0 (31)	34.0 ± 27.6 (49)	2.4 ± 2.8 (3)^d^

*Data are expressed as mean ± SD, and non-normally distributed data are expressed as the median (interquartile range). Different superscript letters a, b, c, and d denote significant differences (*p* < 0.05). GV, germinal vesicle; GVBD, germinal vesicle breakdown; M I, metaphase I; M II, metaphase II; Mdivi-1, mitochondrial division inhibitor 1.*

### Mdivi-1 Targets Mitochondrial Functions and Oxidative Stress in Porcine Oocytes

**FIGURE 3:**
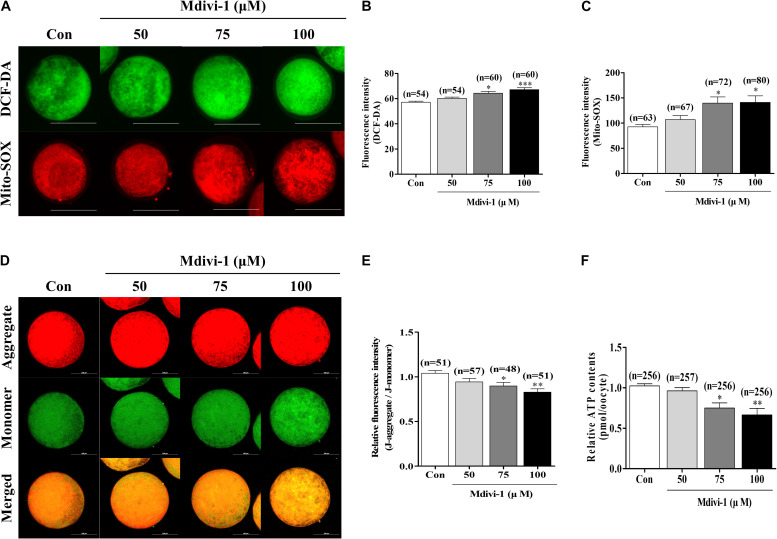
Effects of mitochondrial division inhibitor 1 (Mdivi-1) exposure on mitochondrial functions in mature porcine oocytes. **(A–C)** Representative images of intracellular and mitochondrial reactive oxygen species (ROS) levels in control, 50, 75, or 100 μM Mdivi-1-treated oocytes. Green: intracellular ROS; red: mitochondria-derived superoxide. Scale bar: 200 μm. **(D,E)** Typical images of mitochondrial membrane potential (MMP) in the control and Mdivi-1 (50, 75, and 100 μM) groups. Scale bar: 100 μm. **(F)** Relative ATP contents of mature porcine oocytes from various Mdivi-1-treated groups. Data in the bar graph represent the mean ± SD of three independent experiments (^∗^*p* < 0.05; ^∗∗^*p* < 0.01; ^∗∗∗^*p* < 0.001).

To investigate oxidative stress using Mdivi-1, we investigated intracellular and mitochondrial ROS using DCF-DA and Mito-SOX staining (Figures 3A–C). Both intracellular and mitochondrial ROS levels were significantly higher in porcine oocytes after treatment with 75 or 100 μM Mdivi-1 than those in the control group. We measured mitochondrial membrane potential (MMP) and ATP content using JC-1 staining and an ATP determination kit to confirm mitochondrial functions (Figures 3D–F). MMP was significantly decreased in the 75 and 100 μM Mdivi-1-treated groups compared to that in the control group (Figure 3E). As a result, the ATP production capacity of porcine oocytes was significantly attenuated (Figure 3F). These disturbances in MMP were in line with the reduced ATP production observed in 75 μM Mdivi-1-treated porcine oocytes. The results described above were in agreement with the observed decline in mitochondrial fission (Figure 2); mitochondrial dysfunction was observed in cells treated with 75 μM Mdivi-1. To confirm the half maximal inhibitory concentration (IC_50_), we confirmed the oocyte survival rate by Mdivi-1 treatment using previous IVM data. The valid concentration of Mdivi-1 was approximately 78 μM (Supplementary Figure 1). Based on these results, we optimized the Mdivi-1 concentration to 75 μM, which was then used in subsequent experiments.

### Mdivi-1 Exposure Impairs Spindle Assembly and Location of the Mitochondria Around the Nucleus in Porcine Oocytes

**FIGURE 4:**
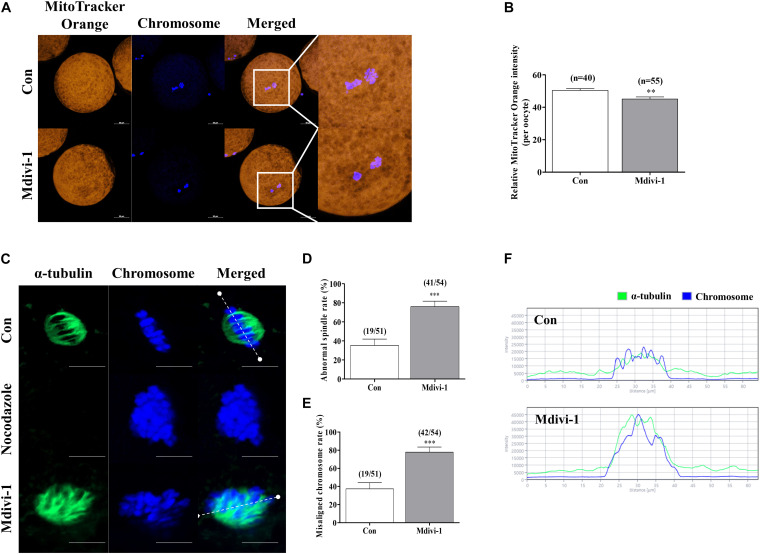
Effects of mitochondrial division inhibitor 1 (Mdivi-1) on spindle assembly in mature porcine oocytes. **(A,B)** Representative images of mitochondrial activation in the control and Mdivi-1-treated groups. Orange: mitochondria; blue: chromosome. Scale bar: 50 μm. **(B)** Quantitative analysis of mitochondria activation in the control and Mdivi-1-treated groups. **(C)** Representative images of spindle morphology and chromosome alignment in the control and Mdivi-1-treated groups. Green: α-tubulin; blue: chromosome. Scale bar: 10 μm. Abnormal spindle **(D)** and misaligned chromosome **(E)** rates of mature oocytes in the control and Mdivi-1-treated groups. **(F)** Arrangement of spindle assembly in the control and Mdivi-1-treated groups. Green: α-tubulin; blue: chromosome. Data represent the mean ± SD of three biological replicates (^∗∗^*p* < 0.01; ^∗∗∗^*p* < 0.001).

The mitochondrial location around the nucleus influences spindle assembly during meiotic maturation in mammalian oocytes (Ding et al., 2020). Therefore, we investigated the changes in mitochondrial location by Mdivi-1 using MitoTracker Orange staining. As a result, the numbers of mitochondria in Mdivi-1-treated groups were conspicuously decreased at the perinuclear site, and fluorescence intensity also declined (Figures 4A,B). Next, we compared spindle abnormalities in oocytes cultured with or without 75 μM Mdivi-1 (Figure 4C). Nocodazole is an antineoplastic agent that interferes with microtubule polymerization and was used as a negative control to confirm the inhibition of the formation of spindle assembly. We found that the percentage of abnormal spindles in porcine oocytes was significantly increased in the 75 μM Mdivi-1-treated group compared to that in the control group (Figures 4D,E). We measured the arrangement of chromosomes and the shape of α-tubulin using the Zen 2.3 (Blue edition) software based on the white dotted line in Figure 4F. The chromosome and α-tubulin form in the control group were shown to be arranged at regular intervals of spindle assembly. In the Mdivi-1-treated group, there were no relatively regular intervals and alignment. No microtubules due to α-tubulin staining were observed in the nocodazole-treated oocytes used as the negative control group for the spindle assembly. In control oocytes, nuclei and α-tubulin were distributed and combined with nuclei at regular intervals. In contrast, nuclei and α-tubulin destruction was observed in the spindle arrangements during spindle assembly in Mdivi-1-treated oocytes. These results showed that Mdivi-1 exposure is directly involved in spindle assembly during porcine meiotic maturation.

### Mdivi-1 Impairs Chromosome and Tubulin Formation-Related Factor Expression in Porcine Oocytes

**FIGURE 5:**
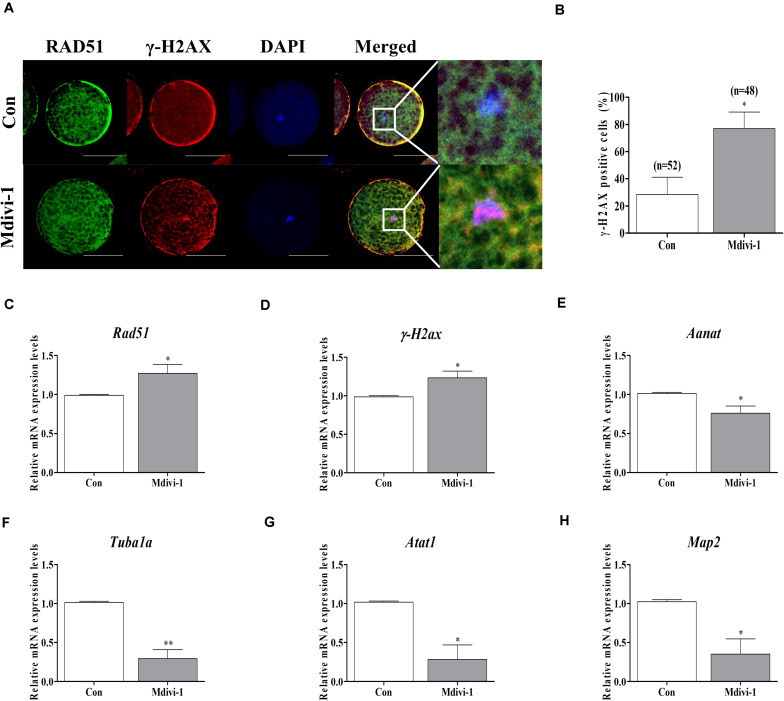
Effects of mitochondrial division inhibitor 1 (Mdivi-1) on the DNA damage and tubulin formation-related genes in mature porcine oocytes. **(A)** Detection of RAD51 (green fluorescence), γ-H2AX (red fluorescence), and chromosome (blue fluorescence) expression using immunofluorescence (IF) staining in mature porcine oocytes. Scale bar: 50 μm. **(B)** Percentage of γ-H2AX-positive cell ratios in mature porcine oocytes. **(C,D)** Expression levels of DNA damage-related genes (*Rad51* and *γ-H2ax*) after Mdivi-1 treatment in mature porcine oocytes. **(E)** The expression level of melatonin synthesis enzyme gene (*Aanat*) after Mdivi-1 treatment in mature porcine oocytes. **(F–H)** Expression levels of tubulin formation-related genes (*Tuba1a*, *Atat1*, and *Map2*) after Mdivi-1 treatment in mature porcine oocytes. Data in the bar graph represent the mean ± SEM of three independent experiments (^∗^*p* < 0.05; ^∗∗^*p* < 0.01).

To confirm direct damage to the spindle assembly by Mdivi-1, we investigated the protein levels of RAD51 and γ-H2AX using immunofluorescence (IF) staining and the mRNA levels of tubulin-related genes using qPCR in mature oocytes. We conducted double staining of RAD51 and γ-H2AX in all mature oocytes. The immunostaining images showed that protein expressions of RAD51 (green fluorescence) and γ-H2AX (red fluorescence) were merged with nuclear (blue fluorescence) in mature porcine oocytes (Figure 5A). Moreover, detection of γ-H2AX-positive cell rates in the Mdivi-1-treated group was higher than that in the control group. The mRNA levels of the DNA repair genes *Rad51* and *γ-H2ax* were significantly increased in Mdivi-1-treated oocytes (Figures 5C,D). Interestingly, the mRNA level of *Aanat*, a melatonin synthesis-related gene from mitochondria, was lower in Mdivi-1-treated oocytes than in control oocytes (Figure 5E). As shown in Figures 5F–H, Mdivi-1 (75 μM) administration significantly decreased the levels of tubulin formation-related gene (*Tubula*, *Atat1*, and *Map2*) transcription in mature oocytes. These results demonstrate that Mdivi-1 promotes the deficiency of spindle assembly by disturbing tubulin formation-related gene expression during meiotic maturation of porcine oocytes.

### Melatonin Relieves the Deterioration of Oocyte Quality Following Spindle Assembly Damage by Mdivi-1 During *in vitro* Maturation

**FIGURE 6:**
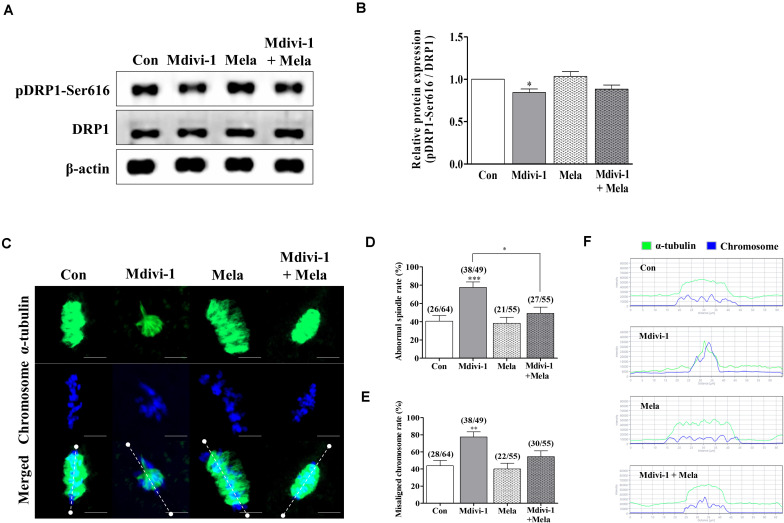
Effect of melatonin on mitochondrial fission and spindle assembly in mature porcine oocytes. **(A,B)** Representative Western blot of the mitochondrial fission [dynamin-related protein 1 phosphorylation at serine 616 (pDRP1-Ser616) and dynamin-related protein 1 (DRP1)] protein levels in the control, mitochondrial division inhibitor 1 (Mdivi-1), melatonin, and Mdivi-1+melatonin groups. Here, β-actin was used as a loading control. The ratios of phosphorylated/total proteins were calculated and plotted in all experimental groups. **(C)** Representative images of spindle morphology and chromosome alignment in the control, Mdivi-1, melatonin, and Mdivi-1 + melatonin groups. Green: α-tubulin; blue: chromosome. Scale bar: 10 μm. Abnormal spindle **(D)** and misaligned chromosome **(E)** rates of mature oocytes in the control, Mdivi-1, melatonin, and Mdivi-1 + melatonin groups. **(F)** Arrangement of spindle assembly in the control, Mdivi-1, melatonin, and Mdivi-1 + melatonin groups. Green: α-tubulin; blue: chromosome. Data represent the mean ± SD/SEM of three biological replicates (^∗^*p* < 0.05; ^∗∗^*p* < 0.01; ^∗∗∗^*p* < 0.001).

Because spindle assembly damage is a major trigger that impairs meiotic maturation, we used melatonin for recovery against disrupted spindle organization. The meiotic maturation rates in the melatonin-treated group after Mdivi-1 pretreatment were restored compared to those in the Mdivi-1-treated group of mature oocytes (Table 3). Numerous reports have shown that melatonin is present in oocytes, which protects against spindle and chromosome damage (Zhang et al., 2017; Lan et al., 2018; An et al., 2019; Li et al., 2019; Chen et al., 2020). We examined pDRP1-Ser616 and DRP1 protein levels in mature oocytes following treatment with 75 μM Mdivi-1 and/or 0.1 μM melatonin (Figures 6A,B). Unfortunately, melatonin did not significantly change mitochondrial fission protein expression in porcine oocytes. Interestingly, the meiotic spindle/chromosome defects in the group treated with 0.1 μM melatonin after Mdivi-1 pretreatment were significantly decreased compared to those in the group treated only with Mdivi-1 (Figures 6C,D). In addition, misaligned chromosome rates were decreased in the melatonin-treated group after Mdivi-1 pretreatment compared with those in the only Mdivi-1-treated group (Figure 6E). As shown by the white dashed line (criterion) in Figure 6C, the abnormal α-tubulin arrangement and chromosomal alignment by Mdivi-1 were restored by melatonin treatment (Figure 6F). Thus, we confirmed that melatonin can restore abnormal spindle formation induced by Mdivi-1 and improve meiotic maturation in porcine oocytes.

**TABLE 3:** Meiotic maturation rate by Mdivi-1 and/or melatonin treatment.

			% of oocytes (n)			
			
Mdivi-1 (75 μM)	Melatonin (100 nM)	No. of oocytes examined	GV	GVBD	M I	M II
–	–	186	2.3 ± 1.9 (5)^a^	2.1 ± 2.1 (3)	17.7 ± 4.7 (33)	77.9 ± 4.3 (145)^a^
+	–	185	10.5 ± 8.2 (22)^b^	7.0 ± 5.2 (14)	37.7 ± 13.1 (67)	44.8 ± 9.4 (82)^b^
–	+	173	2.5 ± 4.2 (6)^a^	2.7 ± 2.5 (5)	16.5 ± 9.6 (30)	78.3 ± 9.2 (132)^a^
+	+	197	4.3 ± 2.1 (9)^a^	0.4 ± 0.8 (1)	24.7 ± 4.5 (47)	70.7 ± 3.7 (140)^a^

*Data are expressed as mean ± SD, and non-normally distributed data are expressed as median (interquartile range). Different superscript letters a and b denote significant differences (*p* < 0.05). GV, germinal vesicle; GVBD, germinal vesicle breakdown; M I, metaphase I; M II, metaphase II; Mdivi-1, mitochondrial division inhibitor 1.*

### Melatonin Reduces Intracellular Reactive Oxygen Species and Mitochondrial Superoxide and Increases Tubulin Formation Protein Levels During Porcine Meiotic Maturation

**FIGURE 7:**
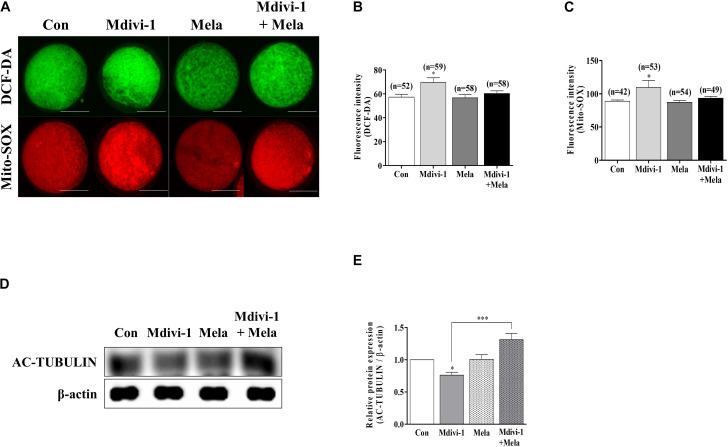
Effects of melatonin on the intracellular and mitochondrial reactive oxygen species (ROS) and tubulin formation-related protein in mature porcine oocytes. **(A–C)** Fluorescence intensity of intracellular (green) and mitochondrial (red) ROS using DCF-DA and Mito-SOX staining. Scale bar: 100 μm. **(D,E)** Representative Western blot of the AC-TUBULIN protein level in the control, mitochondrial division inhibitor 1 (Mdivi-1), melatonin, and Mdivi-1 + melatonin groups. Here, β-actin was used as a loading control. Data in the bar graph represent the mean ± SEM of three independent experiments (^∗^*p* < 0.05; ^∗∗∗^*p* < 0.001).

Melatonin improved porcine oocyte maturation according to the treatment concentration criteria (100 nM) described in our previous study (Park et al., 2018b). To verify the antioxidant ability of melatonin, we treated mature oocytes with melatonin after Mdivi-1 pretreatment and measured intracellular and mitochondrial ROS levels (Figure 7A). The fluorescence intensity of both intracellular (Figure 7B) and mitochondrial (Figure 7C) ROS of mature oocytes was decreased in the melatonin-treated group after Mdivi-1 pretreatment compared to that in the Mdivi-1-treated group. In particular, the expression level of AC-TUBULIN protein was significantly increased in the melatonin-treated group after Mdivi-1 pretreatment compared to that in the control group (Figures 7D,E). The results presented here demonstrate that melatonin suppresses oxidative stress and restores abnormal spindle rates from Mdivi-1-derived damage.

### Restorative Effects of Melatonin Through Chromosome Stabilization and Increasing Tubulin Formation-Related Gene Expression in Mdivi-1-Exposed Oocytes

**FIGURE 8:**
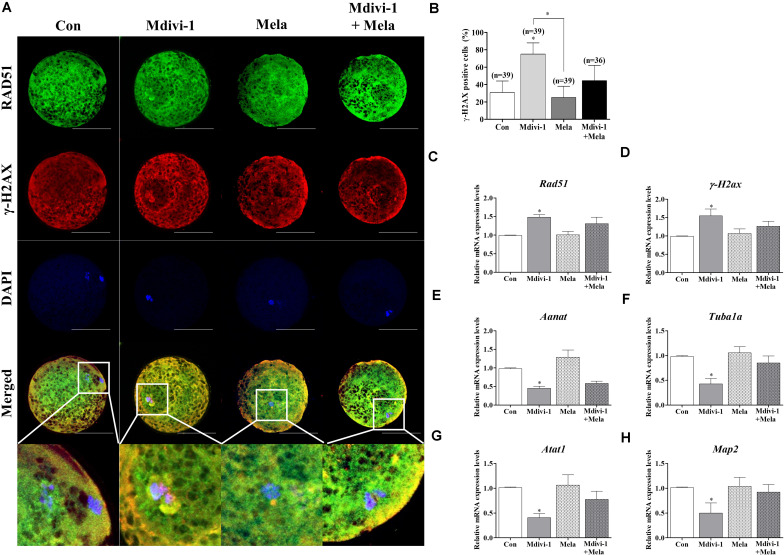
Effects of melatonin on the DNA damage and tubulin formation-related genes in mature porcine oocytes. **(A)** Immunofluorescence of RAD51 and γ-H2AX protein expression of mature porcine oocytes. Scale bar: 100 μm. **(B)** Percentage of γ-H2AX-positive cell ratios in the control, mitochondrial division inhibitor 1 (Mdivi-1), melatonin, and Mdivi-1 + melatonin groups of mature porcine oocytes. **(C,D)** Expression levels of DNA damage-related genes (*Rad51* and *γ-H2ax*) in the control, Mdivi-1, melatonin, and Mdivi-1 + melatonin groups. **(E)** Expression levels of the melatonin synthesis enzyme gene (*Aanat*) in the control, Mdivi-1, melatonin, and Mdivi-1 + melatonin groups. **(F–H)** Expression levels of tubulin formation-related genes (*Tuba1a*, *Atat1*, and *Map2*) in the control, Mdivi-1, melatonin, and Mdivi-1 + melatonin groups. Data in the bar graph represent the mean ± SEM of three independent experiments (^∗^*p* < 0.05).

In order to investigate the reason underlying the positive effect of melatonin on oocyte maturation, the expression of RAD51 and *γ-*H2AX was evaluated (Figure 8A). Treatment with melatonin after Mdivi-1 treatment reduced the *γ-*H2AX-positive cell rate (Figure 8B). The *Rad51* and *γ-H2ax* gene expression in melatonin-treated oocytes after Mdivi-1 treatment was lower than that observed in 75 μM Mdivi-1-treated oocytes (Figures 8C,D). In addition, the expression of *Aanat* in the melatonin-treated group after Mdivi-1 pretreatment was not significantly different from that in the Mdivi-1-treated group (Figure 8E). The expression of *Tuba1a*, *Atat1*, and *Map2* as tubulin formation-related genes was increased in melatonin-treated oocytes after Mdivi-1 supplementation compared with Mdivi-1-treated oocytes (Figures 8F–H). The results suggest that the damaged chromosome and abnormal spindle formation in Mdivi-1-treated oocytes may also be recovered by melatonin.

## Discussion

Melatonin has protective effects on diverse cellular processes, including DNA damage response and spindle assembly; however, the effect of melatonin on DNA damage based on mitochondrial fission in porcine oocytes has not yet been reported. Moreover, the correlation between mitochondrial fission regulation and spindle assembly during IVM of porcine oocytes has not been investigated. Therefore, in this study, we confirmed the protective effects of melatonin on meiotic maturation-related abnormalities in tubulin formation and spindle assembly induced by Mdivi-1 in pigs.

Immature porcine oocytes under IVM are drastically affected by DNA damage, oxidative stress, and mitochondrial mechanisms (Liang et al., 2017b). Mitochondrial morphological and structural changes play a critical role in porcine preimplantation embryo development, including mitochondrial functions and cellular apoptosis (Niu et al., 2019). An increase in the imbalance of mitochondrial fission and fusion is reportedly the primary cause of developmental disorders in *in vitro* culture (IVC) in porcine embryos (Yang et al., 2018). Indeed, excessive mitochondrial fission caused diverse human diseases from neurodegenerative disease to cancer (Serasinghe and Chipuk, 2016). However, changes in mitochondrial morphology during the induction of oocyte maturation have been greatly overlooked in pigs. To confirm this interaction between mitochondrial fission and oocyte maturation, we first detected the mitochondrial morphology and length in porcine oocytes at 0, 22, and 44 h after IVM (Figures 1A–C). It was first confirmed that the length of the mitochondria decreased in porcine oocytes during IVM progression, and elongation was relatively decreased. A previous study reported that mitochondrial fission increases according to the stage of porcine oocyte maturation (Zhang et al., 2020); accordingly, we found an approximately 1.8-fold increase in pDRP1-Ser616 protein expression in 44-h IVM oocytes compared to that in 0-h IVM oocytes (Figures 1D,E). Notably, as shown in Figure 1F, mitochondrial fission protein DRP1 is also observed in the cytoplasm *in vivo* upon follicle development, according to the ovarian follicle size.

Mdivi-1 has been shown to selectively target DRP1 in mammalian cells and suppress its capacity to catalyze GTP hydrolysis (Tanaka and Youle, 2008). Thus, Mdivi-1 can confer selectivity for mitochondrial fission (Rosdah et al., 2016). We found that mitochondria are elongated *via* the fission inhibition response to Mdivi-1 exposure during oocyte maturation, which causes major damage to porcine meiotic maturation (Figure 2). This illustrates another mitochondrial dysfunction regulated by mitochondrial fission (Figure 3). Upon treatment with 75 μM Mdivi-1, both mitochondrial fragmentation length (<1 μm) and DRP1 protein levels were significantly reduced in mature porcine oocytes (Figure 2A). In addition, mitochondria aggregation was significantly increased in mature oocytes treated with 75 and 100 μM Mdivi-1 (Figure 2F). In general, mitochondrial aggregation indicates the irregular distribution of mitochondria in the cytoplasm and is defined by various methods in mammalian oocytes. Mitochondrial aggregation pattern is known to generate in aging oocytes (Soares et al., 2020), and excessive mitochondrial aggregation is an indicator of immature oocytes (Egerszegi et al., 2010). Moreover, that is highly influenced by microtubule stabilization in porcine oocytes (Sun et al., 2001). These results strongly suggest that Mdivi-1 can reduce DRP1 protein levels and induce mitochondrial dysfunction and aggregation in mature porcine oocytes.

To determine mitochondrial activity, we analyzed mature porcine oocytes using MitoTracker Orange staining. Interestingly, we confirmed that Mdivi-1-exposed oocytes had reduced mitochondrial localization around the nucleus (Figure 4A). Nocodazole is a representative microtubule inhibitor that interferes with microtubule polymerization (Tanabe, 2017). Generally, nocodazole disturbs spindle formation in cells and induces cell arrest (Roeles and Tsiavaliaris, 2019). This competence is widely used as an antineoplastic agent. In addition, our results provide evidence for the critical role of DRP1 in spindle assembly and meiotic maturation in porcine oocytes (Figures 4C–F). The migration of mitochondria toward the nuclei assists mitochondria in transferring energy around the nucleus for spindle assembly and microtubule stabilization during porcine oocyte maturation (Sun et al., 2001; Mehta et al., 2019). Microtubules and microfilaments are closely involved with chromosomal dynamics after germinal vesicle breakdown during meiotic maturation of porcine oocytes (Sun and Schatten, 2006). Evidently, sufficient high-energy substrates are required for microtubule polymerization, motor protein activity, and cell cycle-regulating kinases for timely and accurate chromosome segregation (Zhang et al., 2006).

When DNA strand breaks occur, *γ-H2ax* evaluates the damaged site and measures the activity of homologous recombination using *Rad51* (Adam-Zahir et al., 2014). In previous studies, *Rad51* deficiency or knockout caused abnormal spindle assembly in mammalian oocytes (Kim et al., 2016). *γ-H2ax* was well known to DNA damaged gene in oocytes, which has also been used as evidence of abnormal spindle assembly by double-strand breaks (DSBs) in mouse oocyte maturation (Jia et al., 2019; Jiao et al., 2020). We investigated *Rad51* and *γ-H2ax* expression levels to confirm the extent of DNA damage by Mdivi-1 treatment. The predicted expression patterns of *Rad51* and *γ-H2ax* genes are shown in Figures 5A,B. The expressions of *Rad51* and *γ-H2ax* genes in Mdivi-1-treated oocytes were increased, and these findings directly correlated to the results of spindle formation in Figures 4C–F. The *Aanat* gene was recently found to be localized in the mitochondria of oocytes, and melatonin produced from the mitochondria of oocytes has been reported in mice (He et al., 2016; Tan et al., 2016; Yang et al., 2019). According to our results, for the first time, porcine oocyte melatonin production or synthesis was observed by quantitative real-time PCR (qRT-PCR) analysis of the *Aanat* gene (Figure 5C). Furthermore, to our knowledge, no study has examined the change in *Aanat* gene expression for melatonin-synthesizing enzymes by Mdivi-1 in porcine oocytes.

Similarly, whether mitigation of mitochondrial fission by Mdivi-1 leads to changes in microtubule formation-related gene expression or spindle assembly capacity is unknown, especially in the context of oocyte maturation. To observe the correlation with Mdivi-1-dependent spindle assembly damage, we investigated the transcriptional levels of the microtubule-related genes *Tuba1*, *Atat1*, and *Map2* in Mdivi-1-treated oocytes (Figures 5D–F). These microtubule-related genes significantly decreased the expression of mRNA in oocytes treated with 75 μM Mdivi-1. Spindle abnormalities and the disruption of tubulin formation in Mdivi-1-treated oocytes may also be mediated by mitochondrial fission inhibition-dependent reduced meiotic maturation. Our results strongly suggest that Mdivi-1 reduces DRP1 levels and mitochondrial fragmentation in porcine oocytes and disrupts spindle assembly.

Melatonin, now known to be a robust antioxidant that protects against abnormalities in the mitochondrial fragmentation and spindle (Reiter et al., 2016), was subsequently shown to increase IVM and *in vitro* fertilization (IVF) rates in mice (Leem et al., 2019), pigs (Chen et al., 2020), cows (Liang et al., 2017a), and humans (Tamura et al., 2008). Melatonin has been shown to defend against attenuated kinetochore–microtubule attachment stability, disrupting spindle assembly, and chromosome alignment from toxic chemicals such as benzo[ghi]perylene and bisphenol A in mouse oocytes (Zhang et al., 2017; Li et al., 2019). Moreover, melatonin enhances declined meiotic defects by hazardous substances through recovering mitochondrial functions, increasing antioxidant enzyme expression, reducing mitochondria-derived apoptosis, and reducing autophagy levels in porcine oocytes (Park et al., 2018a; Zhang et al., 2018). In this study, the protective function of melatonin against Mdivi-1-mediated spindle assembly damage was studied using qRT-PCR, Western blotting, and α-tubulin IF staining in mature porcine oocytes (Figures 6, 7). Our results showed that melatonin recovered spindle damage and reduced tubulin-related gene expression levels by Mdivi-1 treatment during porcine oocyte maturation. In particular, the protein level of AC-TUBULIN by Mdivi-1 treatment was improved through the protective effects of melatonin (Figures 7A,B). In addition, supplementation with mycotoxin (Lan et al., 2018) and benzo(a)pyrene (Miao et al., 2018) leads to impaired microtubule stability, as shown by the decreased level of AC-TUBULIN response to spindle assembly in mouse oocytes. Based on these results, it is suggested that melatonin has an excellent protective effect on microtubule stability for spindle assembly by interrupting meiotic maturation in Mdivi-1-treated oocytes.

## Conclusion

**FIGURE 9:**
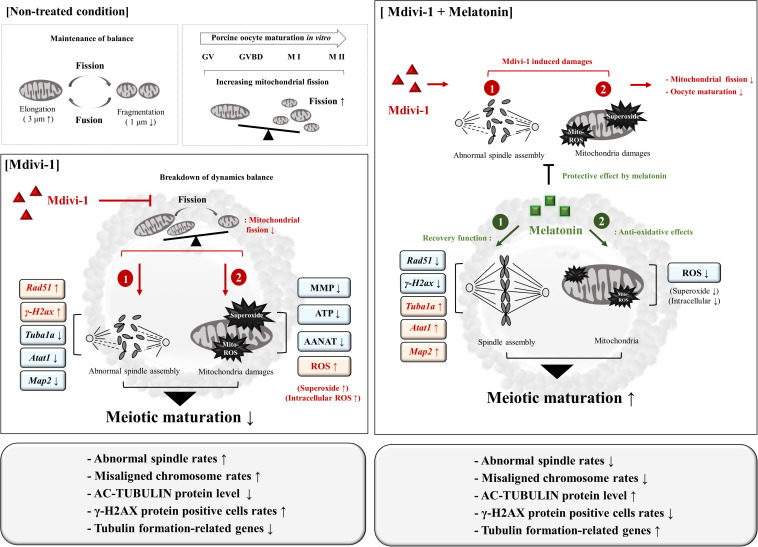
Schematic diagram indicating the suggested protective effects of melatonin on Mdivi-1-inhibited mitochondrial fission in porcine oocytes. In non-treated conditions, mitochondrial fission and fragmentation (<1 μm) increased during oocyte maturation. The Mdivi-1-exposed oocyte increased abnormal spindle assembly (Mdivi-1-induced damage, 1) and mitochondria-derived superoxide assembly (Mdivi-1-induced damage, 2) by blocked mitochondrial fission in pig. Melatonin enhances oocyte maturation capacity against Mdivi-1-mediated damages through restoration of spindle assembly (melatonin recovery effect, 1) and anti-oxidative effector (melatonin recovery effect, 1). Melatonin improves meiotic maturation in Mdivi-1-exposed oocytes as a recovery and protective effect of mitochondrial superoxide and abnormal spindle assembly during IVM.

Mdivi-1 affects embryonic development by inhibiting mitochondrial fission, especially in the female reproductive system. In this study, we explored its effects on porcine oocyte maturation *via* blocking mitochondrial fission and spindle assembly. Moreover, we showed that an appropriate dose of 0.1 μM melatonin was able to protect against disturbances to microtubule formation, abnormal spindle assembly dynamics, and mitochondria-derived superoxide caused by Mdivi-1 (Figure 9). Importantly, while melatonin does not affect the dynamics and structural transformation of mitochondria from Mdivi-1 damage, it showed a recovery effect on the microtubule formation and reduced ROS from mitochondria and microtubule formation during porcine meiotic maturation. Despite our observations that melatonin has an impact on the recovery response to abnormal spindle assembly and mitochondrial ROS in porcine oocytes, its mechanism of action is not involved in mitochondrial fission. Taken together, these results demonstrate that the protective role of melatonin on Mdivi-1-mediated abnormal spindle assembly may be the mechanism by which it rescues oocytes from Mdivi-1-induced toxicity or mitochondrial fission blocking. These novel observations suggest that melatonin may have a protective effect by impacting meiotic maturation from mitochondrial fission-derived damages for the recovery of oocyte maturation. Furthermore, our findings enhance the understanding of oocyte maturation for female infertility mechanisms affecting spindle assembly.

## Data Availability Statement

The datasets presented in this study can be found in online repositories. The names of the repository/repositories and accession number(s) can be found in the article/Supplementary Material.

## Ethics Statement

The animal study was reviewed and approved by Committee of Daegu University.

## Author Contributions

S-GY and H-JP designed the concept of the experiments. S-YJ, J-WB, and G-DH performed the research. S-GY conducted most of the experiments. S-GY and H-JP analyzed the data of the manuscript. S-GY, H-JP, and D-BK wrote and revised this manuscript. All the authors have read and approved the final manuscript.

## Conflict of Interest

The authors declare that the research was conducted in the absence of any commercial or financial relationships that could be construed as a potential conflict of interest.

## References

[B1] Adam-Zahir S. Plowman P. N. Bourton E. C. Sharif F. Parris C. N. (2014). Increased γ-H2AX and Rad51 DNA repair biomarker expression in human cell lines resistant to the chemotherapeutic agents nitrogen mustard and cisplatin. *Chemotherapy* 60 310–320. 10.1159/000430086 26138778

[B2] Adhikari D. Liu K. (2014). The regulation of maturation promoting factor during prophase I arrest and meiotic entry in mammalian oocytes. *Mol. Cell. Endocrinol.* 382 480–487. 10.1016/j.mce.2013.07.027 23916417

[B3] An Q. Peng W. Cheng Y. Lu Z. Zhou C. Zhang Y. (2019). Melatonin supplementation during in vitro maturation of oocyte enhances subsequent development of bovine cloned embryos. *J. Cell. Physiol.* 234 17370–17381. 10.1002/jcp.28357 30786018

[B4] Bennabi I. Terret M.-E. Verlhac M.-H. (2016). Meiotic spindle assembly and chromosome segregation in oocytes. *J. Cell Biol.* 215 611–619. 10.1083/jcb.201607062 27879467 PMC5147004

[B5] Bordt E. A. Clerc P. Roelofs B. A. Saladino A. J. Tretter L. Adam-Vizi V. (2017). The putative Drp1 inhibitor mdivi-1 is a reversible mitochondrial complex I inhibitor that modulates reactive oxygen species. *Developmental cell* 40 583–594.e6. 28350990 10.1016/j.devcel.2017.02.020 PMC5398851

[B6] Chen L. Zhang J. J. Zhang X. Liu X. Zhao S. Huo L. J. (2020). Melatonin protects against defects induced by malathion during porcine oocyte maturation. *J. Cell. Physiol.* 235 2836–2846. 10.1002/jcp.29189 31535366

[B7] Ding M. Feng N. Tang D. Feng J. Li Z. Jia M. (2018). Melatonin prevents D rp1–mediated mitochondrial fission in diabetic hearts through SIRT 1–PGC 1α pathway. *J. Pineal Res.* 65:e12491. 10.1111/jpi.12491 29575122 PMC6099285

[B8] Ding Z.-M. Hua L.-P. Ahmad M. J. Safdar M. Chen F. Wang Y.-S. (2020). Diethylstilbestrol exposure disrupts mouse oocyte meiotic maturation in vitro through affecting spindle assembly and chromosome alignment. *Chemosphere* 249:126182. 10.1016/j.chemosphere.2020.126182 32078850

[B9] Egerszegi I. Alm H. Rátky J. Heleil B. Brüssow K.-P. Torner H. (2010). Meiotic progression, mitochondrial features and fertilisation characteristics of porcine oocytes with different G6PDH activities. *Reproduct. Fert. Dev.* 22 830–838. 10.1071/rd09140 20450835

[B10] Ganem N. J. Pellman D. (2012). Linking abnormal mitosis to the acquisition of DNA damage. *J. Cell Biol.* 199 871–881. 10.1083/jcb.201210040 23229895 PMC3518222

[B11] Garone C. Minczuk M. Tilokani L. Nagashima S. Paupe V. Prudent J. (2018). Mitochondrial dynamics: overview of molecular mechanisms. *Essays Biochem.* 62 341–360. 10.1042/ebc20170104 30030364 PMC6056715

[B12] Han L. Wang H. Li L. Li X. Ge J. Reiter R. J. (2017). Melatonin protects against maternal obesity–associated oxidative stress and meiotic defects in oocytes via the SIRT 3–SOD 2–dependent pathway. *J. Pineal Res.* 63:e12431. 10.1111/jpi.12431 28658527

[B13] He C. Wang J. Zhang Z. Yang M. Li Y. Tian X. (2016). Mitochondria synthesize melatonin to ameliorate its function and improve mice oocyte’s quality under in vitro conditions. *Int. J. Mol. Sci.* 17:939. 10.3390/ijms17060939 27314334 PMC4926472

[B14] Hoshino Y. (2018). Updating the markers for oocyte quality evaluation: intracellular temperature as a new index. *Reproduct. Med. Biol.* 17 434–441. 10.1002/rmb2.12245 30377396 PMC6194278

[B15] Hu C. Huang Y. Li L. (2017). Drp1-dependent mitochondrial fission plays critical roles in physiological and pathological progresses in mammals. *Int. J. Mol. Sci.* 18:144. 10.3390/ijms18010144 28098754 PMC5297777

[B16] Ježek J. Cooper K. F. Strich R. (2018). Reactive oxygen species and mitochondrial dynamics: the yin and yang of mitochondrial dysfunction and cancer progression. *Antioxidants* 7:13. 10.3390/antiox7010013 29337889 PMC5789323

[B17] Jia Z.-Z. Zhang J.-W. Zhou D. Xu D.-Q. Feng X.-Z. (2019). Deltamethrin exposure induces oxidative stress and affects meiotic maturation in mouse oocyte. *Chemosphere* 223 704–713. 10.1016/j.chemosphere.2019.02.092 30802836

[B18] Jiao X. Gonsioroski A. Flaws J. A. Qiao H. (2020). Iodoacetic acid disrupts mouse oocyte maturation by inducing oxidative stress and spindle abnormalities. *Environ. Pollut.* 268:115601. 10.1016/j.envpol.2020.115601 33126034 PMC7746578

[B19] Kim K.-H. Park J.-H. Kim E.-Y. Ko J.-J. Park K.-S. Lee K.-A. (2016). The role of Rad51 in safeguarding mitochondrial activity during the meiotic cell cycle in mammalian oocytes. *Sci. Rep.* 6 1–12. 27677401 10.1038/srep34110 PMC5039699

[B20] Lan M. Han J. Pan M. H. Wan X. Pan Z. N. Sun S. C. (2018). Melatonin protects against defects induced by deoxynivalenol during mouse oocyte maturation. *J. Pineal Res.* 65:e12477. 10.1111/jpi.12477 29453798

[B21] Leem J. Bai G. Y. Kim J. S. Oh J. S. (2019). Melatonin protects mouse oocytes from DNA damage by enhancing nonhomologous end–joining repair. *J. Pineal Res.* 67:e12603. 10.1111/jpi.12603 31370106

[B22] Li W. D. Yu S. Luo S. M. Shen W. Yin S. Sun Q. Y. (2019). Melatonin defends mouse oocyte quality from benzo [ghi] perylene–induced deterioration. *J. Cell. Physiol.* 234 6220–6229. 10.1002/jcp.27351 30317565

[B23] Liang S. Guo J. Choi J.-W. Kim N.-H. Cui X.-S. (2017a). Effect and possible mechanisms of melatonin treatment on the quality and developmental potential of aged bovine oocytes. *Reproduct. Fert. Dev.* 29 1821–1831. 10.1071/rd16223 27780517

[B24] Liang S. Jin Y.-X. Yuan B. Zhang J.-B. Kim N.-H. (2017b). Melatonin enhances the developmental competence of porcine somatic cell nuclear transfer embryos by preventing DNA damage induced by oxidative stress. *Sci. Rep.* 7 1–13. 28894150 10.1038/s41598-017-11161-9 PMC5593819

[B25] López-Doménech G. Covill-Cooke C. Ivankovic D. Halff E. F. Sheehan D. F. Norkett R. (2018). Miro proteins coordinate microtubule–and actin–dependent mitochondrial transport and distribution. *EMBO J.* 37 321–336. 10.15252/embj.201696380 29311115 PMC5793800

[B26] Mayer A. Baran V. Sakakibara Y. Brzakova A. Ferencova I. Motlik J. (2016). DNA damage response during mouse oocyte maturation. *Cell Cycle* 15 546–558. 10.1080/15384101.2015.1128592 26745237 PMC5056612

[B27] Mehta K. Chacko L. A. Chug M. K. Jhunjhunwala S. Ananthanarayanan V. (2019). Association of mitochondria with microtubules inhibits mitochondrial fission by precluding assembly of the fission protein Dnm1. *J. Biol. Chem.* 294 3385–3396. 10.1074/jbc.ra118.006799 30602572 PMC6416417

[B28] Miao Y. Zhou C. Bai Q. Cui Z. ShiYang X. Lu Y. (2018). The protective role of melatonin in porcine oocyte meiotic failure caused by the exposure to benzo (a) pyrene. *Hum. Reproduct.* 33 116–127. 10.1093/humrep/dex331 29112712

[B29] Namgoong S. Kim N.-H. (2018). Meiotic spindle formation in mammalian oocytes: implications for human infertility. *Biol. Reproduct.* 98 153–161. 10.1093/biolre/iox145 29342242

[B30] Niu Y.-J. Nie Z.-W. Shin K.-T. Zhou W. Cui X.-S. (2019). PINK1 regulates mitochondrial morphology via promoting mitochondrial fission in porcine preimplantation embryos. *FASEB J.* 33 7882–7895. 10.1096/fj.201802473r 30897005

[B31] Otera H. Ishihara N. Mihara K. (2013). New insights into the function and regulation of mitochondrial fission. *Biochim. Biophys. Acta (BBA) Mol. Cell Res.* 1833 1256–1268. 10.1016/j.bbamcr.2013.02.002 23434681

[B32] Park H. J. Park J. Y. Kim J. W. Yang S. G. Jung J. M. Kim M. J. (2018b). Melatonin improves the meiotic maturation of porcine oocytes by reducing endoplasmic reticulum stress during in vitro maturation. *J. Pineal Res.* 64:e12458. 10.1111/jpi.12458 29149522 PMC5814851

[B33] Park H.-J. Park S.-Y. Kim J.-W. Yang S.-G. Kim M.-J. Jegal H.-G. (2018a). Melatonin improves oocyte maturation and mitochondrial functions by reducing bisphenol a-derived superoxide in porcine oocytes in vitro. *Int. J. Mol. Sci.* 19:3422. 10.3390/ijms19113422 30384504 PMC6274783

[B34] Park J. Choi H. Min J. S. Park S. J. Kim J. H. Park H. J. (2013). Mitochondrial dynamics modulate the expression of pro–inflammatory mediators in microglial cells. *J. Neurochem.* 127 221–232. 10.1111/jnc.12361 23815397

[B35] Park J.-E. Kim Y.-J. Lee S. G. Kim J. Y. Chung J.-Y. Jeong S.-Y. (2019). Drp1 phosphorylation is indispensable for steroidogenesis in Leydig cells. *Endocrinology* 160 729–743. 10.1210/en.2019-00029 30689811

[B36] Reiter R. J. Mayo J. C. Tan D. X. Sainz R. M. Alatorre–Jimenez M. Qin L. (2016). Melatonin as an antioxidant: under promises but over delivers. *J. Pineal Res.* 61 253–278. 10.1111/jpi.12360 27500468

[B37] Roeles J. Tsiavaliaris G. (2019). Actin-microtubule interplay coordinates spindle assembly in human oocytes. *Nat. Commun.* 10 1–10. 31604948 10.1038/s41467-019-12674-9 PMC6789129

[B38] Romé P. Ohkura H. (2018). A novel microtubule nucleation pathway for meiotic spindle assembly in oocytes. *J. Cell Biol.* 217 3431–3445. 10.1083/jcb.201803172 30087124 PMC6168254

[B39] Rosdah A. A. Holien K. J. Delbridge L. M. Dusting G. J. Lim S. Y. (2016). Mitochondrial fission–a drug target for cytoprotection or cytodestruction? *Pharmacol. Res. Perspect.* 4:e00235. 10.1002/prp2.235 PMC4876145 27433345

[B40] Ruiz A. Alberdi E. Matute C. (2018). Mitochondrial division inhibitor 1 (mdivi-1) protects neurons against excitotoxicity through the modulation of mitochondrial function and intracellular Ca2+ signaling. *Front. Mol. Neurosci.* 11:3. 10.3389/fnmol.2018.00003 PMC5776080 29386996

[B41] Schomerus C. KORF H. W. (2005). Mechanisms regulating melatonin synthesis in the mammalian pineal organ. *Ann. N. Y. Acad. Sci.* 1057 372–383. 10.1196/annals.1356.028 16399907

[B42] Seo B. J. Yoon S. H. Do J. T. (2018). Mitochondrial dynamics in stem cells and differentiation. *Int. J. Mol. Sci.* 19:3893. 10.3390/ijms19123893 30563106 PMC6321186

[B43] Serasinghe M. N. Chipuk J. E. (2016). Mitochondrial fission in human diseases. *Pharmacol. Mitochondria* 240 159–188. 10.1007/164_2016_38 28040850 PMC6388405

[B44] Soares M. Sousa A. P. Fernandes R. Ferreira A. F. Almeida-Santos T. Ramalho-Santos J. (2020). Aging-related mitochondrial alterations in bovine oocytes. *Theriogenology* 157 218–225. 10.1016/j.theriogenology.2020.07.036 32814249

[B45] Sun J. Cui K. Li Z. P. Gao B. Huang B. Liu Q. (2020). Improved early development potence of in vitro fertilization embryos by treatment with tubacin increasing acetylated tubulin of matured porcine oocytes. *Mechanisms Dev.* 164:103631. 10.1016/j.mod.2020.103631 32828904

[B46] Sun Q. Wu G. Lai L. Park K. Cabot R. Cheong H. (2001). Translocation of active mitochondria during pig oocyte maturation, fertilization and early embryo development in vitro. *Reproduction* 122 155–163. 10.1530/reprod/122.1.155 11425340

[B47] Sun Q.-Y. Schatten H. (2006). Regulation of dynamic events by microfilaments during oocyte maturation and fertilization. *Reproduction* 131 193–205. 10.1530/rep.1.00847 16452714

[B48] Tamura H. Nakamura Y. Terron M. P. Flores L. J. Manchester L. C. Tan D.-X. (2008). Melatonin and pregnancy in the human. *Reproductive Toxicol.* 25 291–303. 10.1016/j.reprotox.2008.03.005 18485664

[B49] Tan D.-X. Manchester L. C. Qin L. Reiter R. J. (2016). Melatonin: a mitochondrial targeting molecule involving mitochondrial protection and dynamics. *Int. J. Mol. Sci.* 17:2124. 10.3390/ijms17122124 27999288 PMC5187924

[B50] Tanabe K. (2017). Microtubule depolymerization by kinase inhibitors: unexpected findings of dual inhibitors. *Int. J. Mol. Sci.* 18:2508. 10.3390/ijms18122508 29168788 PMC5751111

[B51] Tanaka A. Youle R. J. (2008). A chemical inhibitor of DRP1 uncouples mitochondrial fission and apoptosis. *Mol. Cell* 29 409–410. 10.1016/j.molcel.2008.02.005 18313377

[B52] Tang F. Pan M.-H. Lu Y. Wan X. Zhang Y. Sun S.-C. (2018). Involvement of Kif4a in spindle formation and chromosome segregation in mouse oocytes. *Aging Dis.* 9:623. 10.14336/ad.2017.0901 30090651 PMC6065292

[B53] Tukur H. A. Aljumaah R. S. Swelum A. A.-A. Alowaimer A. N. Saadeldin I. M. (2020). The making of a competent oocyte-a review of oocyte development and its regulation. *J. Animal Reproduct. Biotechnol.* 35 2–11. 10.12750/jarb.35.1.2

[B54] Udagawa O. Ishihara T. Maeda M. Matsunaga Y. Tsukamoto S. Kawano N. (2014). Mitochondrial fission factor Drp1 maintains oocyte quality via dynamic rearrangement of multiple organelles. *Curr. Biol.* 24 2451–2458. 10.1016/j.cub.2014.08.060 25264261

[B55] Wakai T. Harada Y. Miyado K. Kono T. (2014). Mitochondrial dynamics controlled by mitofusins define organelle positioning and movement during mouse oocyte maturation. *Mol. Hum. Reproduction* 20 1090–1100. 10.1093/molehr/gau064 25113836

[B56] Wloga D. Joachimiak E. Fabczak H. (2017). Tubulin post-translational modifications and microtubule dynamics. *Int. J. Mol. Sci.* 18:2207. 10.3390/ijms18102207 29065455 PMC5666887

[B57] Yang L. Wang Q. Cui M. Li Q. Mu S. Zhao Z. (2020). Effect of melatonin on the in vitro maturation of porcine oocytes, development of parthenogenetically activated embryos, and expression of genes related to the oocyte developmental capability. *Animals* 10:209. 10.3390/ani10020209 32012669 PMC7070577

[B58] Yang M. Tao J. Wu H. Guan S. Liu L. Zhang L. (2019). Aanat knockdown and melatonin supplementation in embryo development: involvement of mitochondrial function and DNA methylation. *Antioxidants Redox Sign.* 30 2050–2065. 10.1089/ars.2018.7555 30343588

[B59] Yang S.-G. Park H.-J. Kim J.-W. Jung J.-M. Kim M.-J. Jegal H.-G. (2018). Mito-TEMPO improves development competence by reducing superoxide in preimplantation porcine embryos. *Sci. Rep.* 8 1–10. 29973637 10.1038/s41598-018-28497-5 PMC6031607

[B60] Yi Z.-Y. Ma X.-S. Liang Q.-X. Zhang T. Xu Z.-Y. Meng T.-G. (2016). Kif2a regulates spindle organization and cell cycle progression in meiotic oocytes. *Sci. Rep.* 6 1–12. 27991495 10.1038/srep38574 PMC5171826

[B61] Youle R. J. Van Der Bliek A. M. (2012). Mitochondrial fission, fusion, and stress. *Science* 337 1062–1065. 10.1126/science.1219855 22936770 PMC4762028

[B62] Zenker J. White M. D. Templin R. Parton R. Thorn-Seshold O. Bissiere S. (2017). A microtubule-organizing center directing intracellular transport in the early mouse embryo. *Science* 357 925–928. 10.1126/science.aam9335 28860385

[B63] Zhang H. Pan Z. Ju J. Xing C. Li X. Shan M. (2020). DRP1 deficiency induces mitochondrial dysfunction and oxidative stress-mediated apoptosis during porcine oocyte maturation. *J. Animal Sci. Biotechnol.* 11 1–10. 10.1016/j.theriogenology.2021.01.011 32782788 PMC7409671

[B64] Zhang M. Dai X. Lu Y. Miao Y. Zhou C. Cui Z. (2017). Melatonin protects oocyte quality from Bisphenol A–induced deterioration in the mouse. *J. Pineal Res.* 62:e12396. 10.1111/jpi.12396 28178360

[B65] Zhang X. Wu X. Q. Lu S. Guo Y. L. Ma X. (2006). Deficit of mitochondria-derived ATP during oxidative stress impairs mouse MII oocyte spindles. *Cell Res.* 16 841–850. 10.1038/sj.cr.7310095 16983401

[B66] Zhang Y. Wang T. Lan M. Zang X.-W. Li Y.-L. Cui X.-S. (2018). Melatonin protects oocytes from MEHP exposure-induced meiosis defects in porcine. *Biol. Reproduction* 98 286–298. 10.1093/biolre/iox185 29373650

