# Supplementary material for: Melatonin Protects Against Mdivi-1-Induced Abnormal Spindle Assembly and Mitochondrial Superoxide Production During Porcine Oocyte Maturation
Source: Front Cell Dev Biol. 2021 Jul 8;9:693969. doi: 10.3389/fcell.2021.693969 (PMC8297652; doi:10.3389/fcell.2021.693969)
Supplement: Supplementary file 1 [file Data_Sheet_1.docx]

**Supplementary Figure**


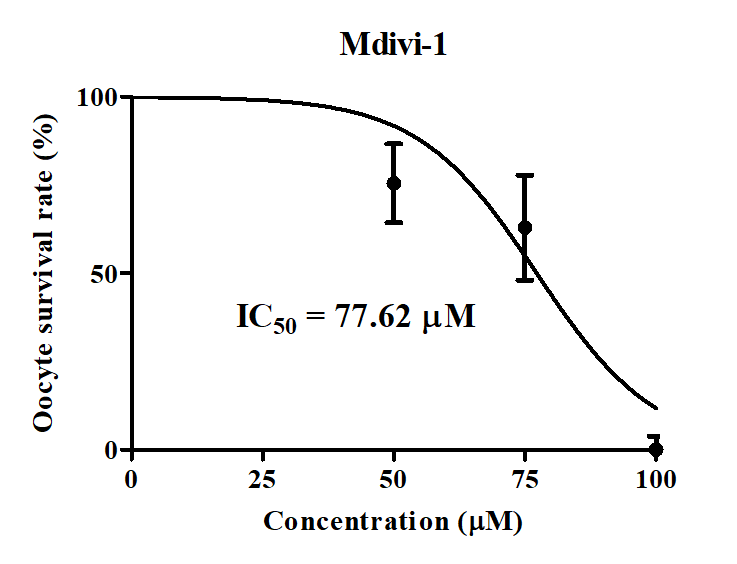


**Supplementary Figure 1.** Measurement of the half maximal inhibitory concentration (IC_50_) according to the dose of Mdivi-1. The graph represents the rates of oocyte meiotic maturation (Table 2) and was generated using GraphPad 5.0 software. IC_50_ = 77.62 μM Mdivi-1. Data represent the mean ± SD of three biological replicates.
